# Association between off-hour presentation and endotracheal-intubation-related adverse events in trauma patients with a predicted difficult airway: A historical cohort study at a community emergency department in Japan

**DOI:** 10.1186/s13049-016-0296-2

**Published:** 2016-08-30

**Authors:** Yuko Ono, Takuya Sugiyama, Yasuyuki Chida, Tetsuya Sato, Hiroaki Kikuchi, Daiji Suzuki, Masakazu Ikeda, Koichi Tanigawa, Kazuaki Shinohara

**Affiliations:** 1 Department of Anesthesiology, Ohta General Hospital Foundation, Ohta Nishinouchi Hospital, 2-5-20 Nishinouchi, Koriyama, Fukushima 963-8558 Japan; 2 Emergency and Critical Care Medical Center, Fukushima Medical University Hospital, 1 Hikarigaoka, Fukushima, 960-1295 Japan; 3 Department of Head and Neck Surgery, Ohta General Hospital Foundation, Ohta Nishinouchi Hospital, 2-5-20 Nishinouchi, Koriyama, Fukushima 963-8558 Japan; 4 Department of Otolaryngology, Fukushima Medical University, 1 Hikarigaoka, Fukushima, 960-1295 Japan; 5 Fukushima Medical University, 1 Hikarigaoka, Fukushima, 960-1295 Japan; 6 Fukushima Global Medical Science Center, 1 Hikarigaoka, Fukushima, 960-1295 Japan

**Keywords:** Airway management, Comminuted facial trauma, Difficult airway management, Fatal complication, Inhalational burn, Penetrating neck injury

## Abstract

**Background:**

A reduction in medical staff such as occurs in hospitals during nights and weekends (off hours) is associated with a worse outcome in patients with several unanticipated critical conditions. Although difficult airway management (DAM) requires the simultaneous assistance of several appropriately trained medical caregivers, data are scarce regarding the association between off-hour presentation and endotracheal intubation (ETI)-related adverse events, especially in the trauma population. The aim of this study was to determine whether off-hour presentation was associated with ETI complications in injured patients with a predicted difficult airway.

**Methods:**

This historical cohort study was conducted at a Japanese community emergency department (ED). All patients with inhalation burn, comminuted facial trauma (Abbreviated Injury Scale Score Face ≥3), and penetrating neck injury who underwent ETI from January 2007 to January 2016 in our ED were included. Primary exposure was off-hour presentation, defined as the period from 6:01 PM to 8:00 AM weekdays plus the entire weekend. The primary outcome measure was the occurrence of an ETI-related adverse event, including hypoxemia, unrecognized esophageal intubation, regurgitation, cardiac arrest, ETI failure rescued by emergency surgical airway, cuff leak, and mainstem bronchus intubation.

**Results:**

Of the 123 patients, 75 (61.0 %) were intubated during off hours. Crude analysis showed that off-hour presentation was significantly associated with an increased risk of ETI-related adverse events [odds ratio (OR), 2.5; 95 % confidence interval (CI), 1.1–5.6; *p* = 0.033]. The increased risk remained significant after adjusting for potential confounders, including operator being an anesthesiologist, use of a paralytic agent, and injury severity score (OR, 3.0; 95 % CI, 1.1–8.4; *p* = 0.034).

**Conclusions:**

In this study, off-hour presentation was independently associated with ETI-related adverse events in trauma patients with a predicted difficult airway. These data imply the need for more attentive hospital care during nights and weekends.

**Electronic supplementary material:**

The online version of this article (doi:10.1186/s13049-016-0296-2) contains supplementary material, which is available to authorized users.

## Background

Human resources play a very important role in difficult airway management (DAM). In DAM algorithms advocated by several professional anesthesiology societies, the “call for help” is a common first step and the most important action [1–3]. Jaber et al. recently reported that the presence of backup staff was independently associated with a reduced risk of complications related endotracheal intubation (ETI) performed in the intensive care unit (ICU) [4].

A shortage of medical staff is one of the most serious healthcare problems in Japan, especially in the provinces [5]. Moreover, at most medical institutions, including our own, staffing levels dramatically decrease during off hours; i.e., nights and weekends. At these times, not only is in-house, experienced back-up coverage less available [6], but staff performance may be impaired because of fatigue and disrupted circadian rhythms [7]. Off-hour presentation was reported to be associated with adverse outcomes in patients with unanticipated critical conditions requiring aggressive intervention, including cardiac arrest [8], myocardial infarction [9], ruptured aortic aneurysm [10], and stroke [6]. In a previous study, we found that emergency department (ED) presentation during off hours was associated with a longer ED stay, but not an increase in mortality for severely injured patients requiring emergency trauma surgery [5].

ETI is a common and critical intervention in the ED. However, studies on the association between off-hour presentation and ETI-related adverse events in the ED or other settings are limited [11–13], and, to the best of our knowledge, none have examined the association between off-hour presentation and ETI-related complications in trauma patients with a predicted difficult airway, such as those with inhalation burn, comminuted facial trauma, and penetrating neck injury. Because the consequences of DAM can be catastrophic [14–16], such studies are urgently needed. Thus, we sought to clarify whether off-hour presentation was associated with ETI-related adverse events in injured patients with predicted DAM scenarios.

## Methods

### Study design and setting

This was a historical cohort study conducted at Ohta Nishinouchi Hospital, a community hospital in a provincial Japanese city located approximately 200 km north of Tokyo. The hospital serves as a teaching facility and a referral medical center. Annually, it receives over 1200 trauma patients with injuries of varying severity and from areas within a 50-km radius. A more detailed ED census, including ETI occurrence and overall ETI success rate during 2007 to 2016, is available in the Additional file 1.

As a typical community hospital in Japan, the staffing level of our facility declines significantly during off hours. During business hours on weekdays, the facility has more than 120 in-house physicians, including residents, while at night and on weekends there are fewer than 10 in-house physicians. In the ED, during business hours two or three attending ED physicians and two or three residents (postgraduate year 1 or 2) take part in the initial management of trauma patients, but during off hours one attending ED physician and one resident are present. Attending ED physicians are primarily responsible for airway management in severe trauma patients.

As previously described [17, 18], most Japanese EDs including our own operate according to a multispecialty staffing model. For example, in our hospital ED physicians receive their principal training in surgery or anesthesia and then undergo additional training in emergency medicine. Therefore, there are two ETI expertise levels of ED physicians in our facility. ED physicians with a background in surgery have a minimum of 4 years’ clinical experience. Before starting ED shifts, they must complete at least 3 months of airway management training in the operating room and experience at least 60 ETIs. ED physicians with a background in anesthesiology have a minimum of 4 years’ clinical experience before entering the ED shift. They are involved in approximately 300 ETIs annually involving the full spectrum of difficult airway situations such as head and neck surgery, pediatric anesthesia, and differential lung ventilation.

If DAM is encountered, backups provided by, for example, head and neck surgeons and anesthesiologists are immediately available in-house during business hours, but in most cases only from outside the hospital during off hours. A standard operating procedure for ETI [19], such as unified DAM equipment set-up, pre-ETI assessment, and post-intubation care with end-tidal CO_2_ detection, has not yet been established in our ED.

### Participants and data sources

The study was approved by the Institutional Review Board at Ohta Nishinouchi Hospital (No.14_H27) and included all trauma patients with a predicted difficult airway who underwent emergency ETI in our ED from January 1, 2007, to January 1, 2016. Injured patients with predicted difficult intubations were defined as those with 1) inhalation burn; 2) penetrating neck injury, and 3) comminuted facial trauma [Abbreviated Injury Scale Score (AIS) Face ≥3]. The exclusion criteria were: patients who received ongoing cardiopulmonary resuscitation on ED arrival; pediatric patients (age < 18 years); patients whose initial ETI attempt was by a junior resident; and patients who underwent nasal intubation, surgical airway as an initial intubation attempt, or an alternative technique such as video laryngoscopy, and fiber optic intubation.

Data were collected from an electronic database on trauma and quality assurance, ED records, and nursing records. Our ED maintains a rigorous peer-review process to ensure the quality of our trauma practice. Life-threatening ETI-associated adverse events, such as cardiac arrest after ETI attempt, failed intubation salvaged by emergency surgical airway, and esophageal intubation with delayed recognition, occurring in our ED are peer reviewed, confirmed by experienced ED physicians, and recorded in the quality assurance database without delay. This database also records injury severity as represented by the AIS of each body region, the Injury Severity Score (ISS) [20, 21], the Revised Trauma Score (RTS) [22, 23], and the probability of survival (Ps), which is based on the Trauma and Injury-Severity Scores method [24–26]. ISS, RTS, and Ps were scored without delay by one of the authors (KS). The ISS was scored based on anatomical information obtained by physical examination, X-ray, computed tomography, and operative findings. RTS was scored based on vital signs measured immediately after ED admission. Comorbidity was described using the Charlson comorbidity index [27, 28], which is a weighted index of the number of serious comorbidities on a scale from 0 (no comorbid disease) to 8 (serious comorbid disease). Charlson comorbidity index was scored retrospectively by author YO. Our department uses a structured ED record that includes the patient’s age, sex, initial vital signs on ED arrival, time course, medical history, detailed history of the present condition, physical examination, laboratory data, radiological findings, final diagnosis, and any adverse events in the ED. All physicians who participate in the management of ED patients are required to complete the form immediately, and an ED director at our hospital (KS) checks all medical records to verify the completeness and reliability of the data at the earliest possible opportunity. As specified in several professional anesthesiology societies’ guidelines [1–3], documentation of details of the airway difficulty in the ED record is strongly encouraged in our ED to provide relevant information on ETI-related adverse events. Nursing records include information on the laryngoscopist, the number of ETI attempts, the medication used to facilitate ETI, and the patient’s vital signs after ETI attempts. Correct endotracheal tube placement is verified based on clinical findings, such as tube fogging, chest rise, and auscultation, with secondary confirmation by capnometry performed at the discretion of the attending ED physicians. In our ED, a chest X-ray or computed tomography scan is routinely taken after tube placement to detect mainstem bronchus intubation.

### Exposures and outcome measurement

The primary exposure was presentation to the ED during off hours. For consistency with our own studies and those of other researchers [5, 29], off hours were defined as the period from 6:01 PM to 8:00 AM weekdays plus the entire weekend, and business hours as the period from 8:01 AM to 6:00 PM weekdays.

The primary outcome measure was a composite of ETI-associated adverse events, including hypoxemia, esophageal intubation with delayed recognition, recorded regurgitation, cardiac arrest immediately after ETI attempt, ETI failure rescued by emergency surgical airway, cuff leak requiring intubation, and mainstem bronchus intubation. *Hypoxemia* was defined as a decline in pulse oximetry saturation > 10 % from baseline during ETI attempts, not as a result of esophageal intubation [30]. *Esophageal intubation with delayed recognition* was defined as misplacement of the endotracheal tube in the upper esophagus or hypopharynx, with time elapsed and desaturation (>10 % decline in saturation on pulse oximetry) [30–32]. *Recorded regurgitation* was defined as the immediate peri-induction of gastric contents at the glottis opening or in the endotracheal tube, clearly documented in the ED or in the nursing records [30–32]. *Cardiac arrest immediately after ETI attempt* included asystole, bradycardia, or dysrhythmia without measurable blood pressure and requiring cardiopulmonary resuscitation during or immediately after the ETI attempt [31, 32]. If ETI was impossible after anesthesia induction and required salvage surgical technique, then the event was classified as *ETI failure rescued by emergency surgical airway* [16]. *Mainstem bronchus intubation* was determined on a chest X-ray or computed tomography scan taken immediately after the ETI procedure. Some previous studies have included hemodynamic parameters, such as hypertension and hypotension in their definition of ETI-related complications [11, 13–15, 30–32]. However, for consistency with the more recent literature [12, 33], we chose not to include hemodynamic data because it is difficult to distinguish ETI-related hemodynamic perturbations from underlying trauma-based etiologies. To minimize bias, information on the presentation time was masked during the verification and analysis of ETI-related complications.

### Statistical analysis

First, the differences in the baseline clinical characteristics of trauma patients with a predicted difficult airway who were treated during off hours vs. business hours were evaluated. Differences in continuous variables, including age and vital signs, between the two groups were compared using Student’s *t*-test after first verifying the normal distribution of the data by the Shapiro–Wilk test; otherwise, the Mann–Whitney *U*-test was used. Differences in the Glasgow Coma Scale score, AIS, and ISS between the two groups were compared using the Mann-Whitney *U*-test. Differences in categorical variables, including sex, mortality, indication for ETI, and ETI method used, between the two groups were compared using Fisher’s exact test.

A crude odds ratio (OR) was then calculated to estimate the relative risks of ETI-related adverse in injured patients during off hours, using a 2 × 2 contingency table. Fisher’s exact test was used to produce *p* values.

Potential confounders, including operator’s specialty (anesthesiologist or not), ISS, and use of a paralytic agent were adjusted for using multivariate logistic regression analysis, yielding an adjusted OR for ETI-related adverse events for off-hour presentation as first exposure. A set of potential confounders was chosen based on previous information (operator with anesthesiology background [15, 30, 34, 35], anatomical severity [20, 21], and use of paralytic agent [36–39]).

To determine whether ETI-related complications increased the mortality of injured patients, independent of age and ISS, a second logistic regression model was constructed.

A variance-inflation factor was used to detect multicollinearity and the model’s fit was verified using the Hosmer–Lemeshow test. All statistical analyses were performed using SPSS Statistics for Windows, Version 22.0 (IBM Corp., Armonk, NY, USA). A *p* value < 0.05 was considered to indicate statistical significance.

### Sample size

During the planning of this study, a power analysis was performed using G*Power 3 for Windows (Heinrich Heine University, Düsseldorf, Germany). To our knowledge, no previous study has examined the association between the presentation time and ETI-related adverse events in the population of interest in this work; therefore, we referred to previous studies conducted in similar settings (ICUs receiving mixed critical cases, including trauma, acute-onset medical conditions, and post-operative patients [4, 11, 15]) to calculate the effect size. Based on the assumption that 40 % of the patients who underwent emergency ETI during business hours experienced adverse events [4, 11, 15], a sample size of 73 patients per group would provide 80 % statistical power to detect a 20 % primary outcome difference at a two-tailed α of 0.05.

## Results

**Fig. 1:**
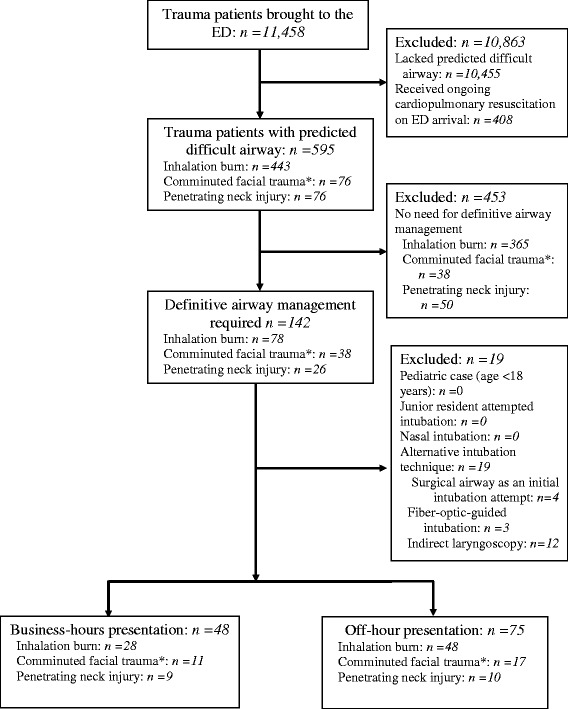
Injured patients with a predicted difficult airway who underwent endotracheal intubation (ETI) in the emergency department (ED).* Patient with an Abbreviated Injury Scale (AIS) Face score ≥ 3

During the 108-month study period, 11,458 trauma patients were brought to the ED, of whom 595 had a predicted difficult airway, including 142 who required ETI in the ED. The 19 patients who underwent initial airway management by emergency surgical airway or an alternative intubation technique were excluded. The remaining 123 patients were included in the analysis. Of these patients, 48 (39.0 %) were admitted to the ED during business hours and 75 (61.0 %) during off hours (Fig. 1). There were no missing data.

Table 1 compares the clinical demographics by presentation time. The median age of patients requiring ETI was 59 years, and approximately two-thirds were male. Other than AIS Chest and AIS External, there were no differences in the clinical characteristics of the patients seen during business hours and off hours, including anatomical and physiological severities, comorbidity score, trauma etiologies, and survival.

**Table 1:** Demographic characteristics of injured patients who had a predicted difficult airway: business hours^a^ vs. off hours^b^

Variable	All (*n* = 123)	Business hours (*n* = 48)	Off hours (*n* = 75)	*p* value
Age (years)	59.0 (34.0–70.0)	57.0 (32.0–65.5)	59.0 (39.5–70.5)	0.42
Male, n (%)	85 (69.1)	36 (75.0)	49 (65.3)	0.32
Glasgow Coma Scale score	13.0 (9.0–15.0)	13.0 (10.8–15.0)	13.0 (8.5–15.0)	0.97
Initial vital signs recorded in the ED				
Systolic blood pressure (mmHg)	130.0 (107.0–163.0)	142.0 (113.5–170.0)	127.5 (106.3–149.5)	0.23
Heart rate (beats/min)	100.0 (80.0–120.0)	92.0 (79.8–120.0)	103.0 (82.5–123.5)	0.35
Respiratory rate (breaths/min)	20.0 (18.0–30.0)	20.0 (16.0–30.0)	20.0 (18.0–30.0)	0.51
Pulse oximetry saturation	100.0 (98.0–100.0)	100.0 (99.0–100.0)	100.0 (98.0–100.0)	0.53
ISS	26.0 (16.0–41.0)	26.0 (16.3–35.0)	26.0 (16.5–41.0)	0.96
AIS				
Head or neck	0 (0–2.0)	0 (0–2.0)	0 (0–3.0)	0.86
Face	0 (0–1.0)	0 (0–0)	0 (0–1.5)	0.55
Chest	3.0 (0–4.0)	0 (0–3.3)	3.0 (0–5.0)	0.033
Abdomen or pelvic contents	0 (0–0)	0 (0–0)	0 (0–0)	0.54
Extremities or pelvic girdle	0 (0–1.0)	0 (0–0.3)	0 (0–1.0)	0.21
External	1.0 (0–5.0)	2.0 (0–5.0)	0 (0–4.0)	0.031
RTS	7.8 (5.9–7.8)	7.8 (6.2–7.8)	7.8 (5.7–7.8)	0.79
Ps	0.91 (0.62–0.97)	0.90 (0.65–0.98)	0.91 (0.62–0.96)	0.68
Charlson Comorbidity Index	0 (0–1.0)	0 (0–1.0)	0 (0–1.0)	1.00
Suicidal attempt, n (%)	45 (36.6)	21 (43.8)	24 (32.0)	0.25
Trauma etiology				
Inhalation burn, n (%)	76 (61.8)	28 (58.3)	48 (64.0)	0.57
Comminuted facial trauma^c^, n (%)	28 (22.8)	11 (22.9)	17 (22.7)	1.00
Penetrating neck injury, n (%)	19 (15.4)	9 (18.8)	10 (13.3)	0.45
Survival (%)	70 (56.9)	27 (56.3)	43 (57.3)	1.00

Data are expressed as the median (interquartile range) or n (%)

*AIS* Abbreviated Injury Scale Score, *ED* emergency department, *ISS* Injury Severity Score, *Ps* probability of survival, *RTS* Revised Trauma Score

^a^8:01 AM to 6:00 PM weekdays

^b^6:01 PM to 8:00 AM weekdays plus all weekend hours

^c^AIS Face ≥3

Table 2 summarizes the characteristics of emergency airway management in patients with a predicted difficult airway. An anesthesiologist is more likely to have performed ETI during business hours (*p* = 0.003). There were no differences between the time of presentation and either the ETI method or the induction agent used.

**Table 2:** Characteristics of airway management in injured patients with a predicted difficult airway: business hours^a^ vs. off hours^b^

	All (*n* = 123)	Business hours (*n* = 48)	Off hours (*n* = 75)	*p* Value
Three or more ETI attempts, n (%)	9 (7.3)	4 (8.3)	5 (6.7)	0.74
An anesthesiologist performed ETI, n (%)	63 (51.2)	33 (68.8)	30 (40.0)	0.003
ETI method, n (%)				
Without medication	23 (18.7)	10 (20.8)	13 (17.3)	0.64
Sedative/Analgesic only	28 (22.8)	9 (18.8)	19 (25.3)	0.51
Paralytic agent only	6 (4.9)	2 (4.2)	4 (5.3)	1.00
Sedative/analgesic + paralytic agent	66 (53.7)	27 (56.3)	39 (52.0)	0.71
Sedative^c^, n (%)				
No sedative	36 (29.3)	14 (29.2)	22 (29.3)	1.00
Midazolam	80 (65.0)	32 (66.7)	48 (64.0)	0.85
Propofol	8 (6.5)	3 (6.3)	5 (6.7)	1.00
Ketamine	2 (1.6)	0 (0)	2 (2.7)	0.52
Thiopental	2 (1.6)	0 (0)	2 (2.7)	0.52
Analgesic^c^, n (%)				
No analgesic	78 (63.4)	32 (66.7)	46 (61.3)	0.57
Fentanyl	36 (29.3)	14 (29.2)	22 (29.3)	1.00
Morphine	1 (0.81)	1 (2.1)	0 (0)	0.39
Buprenorphine	3 (2.4)	1 (2.1)	2 (2.7)	1.00
Pentazocine	3 (2.4)	0 (0)	3 (4.0)	0.28
Butorphanol	2 (1.6)	0 (0)	2 (2.7)	0.52
Lidocaine	1 (0.81)	0 (0)	1 (1.3)	1.00
Paralytic agent, n (%)				
No paralytic agent	54 (43.9)	20 (41.7)	34 (45.3)	0.71
Vecuronium	41 (33.3)	18 (37.5)	23 (30.7)	0.44
Rocuronium	28 (22.8)	10 (20.8)	18 (24.0)	0.83
Succinylcholine	0 (0)	0 (0)	0 (0)	N/A

*ETI* endotracheal intubation, *N*/*A* not available

^a^8:01 AM to 6:00 PM weekdays

^b^6:01 PM to 8:00 AM weekdays plus all weekend hours

^c^More than one drug may have been used to facilitate ETI

Table 3 shows the detailed distribution and crude analysis of ETI-related adverse events during business hours and off hours. Overall, 35.0 % of the study population experienced ETI-associated adverse events, among which mainstem bronchus intubation was the most common. Patients who received emergency ETI during off hours were at increased risk for a composite of adverse events [crude OR, 2.5; 95 % confidence interval (CI), 1.1–5.6; *p* = 0.033]. The increase in absolute risk associated with off–hour presentation for all adverse events was 19.8 % (95 % CI, 2.5–33.9).

**Table 3:** Detailed distribution and crude analysis of emergency ETI-related adverse events in injured patients with a predicted difficult airway: business hours^a^ vs. off hours^b^

Variable	All (*n* = 123)	Business hours (*n* = 48)	Off hours (*n* = 75)	Crude OR (95 % CI)	*p* value
All adverse events, n (%)^c^	43 (35.0)	11 (22.9)	32 (42.7)	2.5 (1.1–5.6)	0.033
Hypoxemia	7 (5.7)	2 (4.2)	5 (6.7)	1.6 (0.3–8.8)	0.70
Esophageal intubation with delayed recognition	5 (4.1)	0 (0)	5 (6.7)	N/A	0.16
Recorded regurgitation	5 (4.1)	2 (4.2)	3 (4.0)	1.0 (0.2–6.0)	1.00
Cardiac arrest immediately after ETI attempt	5 (4.1)	1 (2.1)	4 (5.3)	2.6 (0.3–24.4)	0.65
ETI failure rescued by emergency surgical airway	8 (6.5)	3 (6.3)	5 (6.7)	1.1 (0.2–4.7)	1.00
Cuff leak requiring reintubation	2 (1.6)	0 (0)	2 (2.7)	N/A	0.52
Mainstem bronchus intubation	11 (8.9)	3 (6.3)	8 (10.7)	1.8 (0.5–7.1)	0.53

*CI* confidence interval, *ETI* endotracheal intubation, *OR* odds ratio

^a^8:01 AM to 6:00 PM weekdays

^b^6:01 PM to 8:00 AM weekdays plus all weekend hours

^c^Patients may have had more than one ETI-related adverse event

The results of the multivariate logistic regression analysis for ETI-related adverse events are shown in Table 4. The increased-risk associated with off-hour presentation remained statistically significant after adjusting for the potential confounding factors of operator being an anesthesiologist, use of a paralytic agent, and ISS (adjusted OR, 3.0; 95 % CI, 1.1–8.4; *p* = 0.034). A second multivariate logistic regression analysis for patient mortality showed that ETI-related complications did not affect mortality, independent of age and ISS (Table 5).

**Table 4:** Multivariate logistic regression model of factors associated with ETI-related adverse events in injured patients with a predicted difficult airway

	Adjusted OR (95 % CI)	*p* Value
ETI during off hours^a^	3.0 (1.1–8.4)	0.034
ETI performed by an anesthesiologist	1.3 (0.5–3.3)	0.54
Use of a paralytic agent	0.7 (0.3–1.7)	0.45
ISS	1.1 (1.0–1.1)	<0.001

*CI* confidence interval, *ETI* endotracheal intubation, *ISS* Injury Severity Score, *OR* odds ratio

^a^6:01 PM to 8:00 AM weekdays plus all weekend hours

**Table 5:** Multivariate logistic regression model of factors associated with mortality in injured patients with a predicted difficult airway

	Adjusted OR (95 % CI)	*p* value
Occurrence of ETI associated adverse events	1.1 (0.4–3.1)	0.85
Age	1.0 (1.0–1.1)	0.004
ISS	1.1 (1.1–1.1)	<0.001

*CI* confidence interval, *ETI* endotracheal intubation, *ISS* Injury Severity Score, *OR* odds ratio

Multicollinearity was not detected (variance-inflation factor < 1.1 for each explanatory variable of each model), and the Hosmer–Lemeshow test verified the good fit (*p* > 0.05) of each logistic regression model.

## Discussion

In this analysis of trauma patients with a predicted difficult airway who were treated at a typical community ED in Japan, severe ETI-related adverse events were common and associated with off-hour presentation. These findings highlight the need for care providers to be more vigilant in treating patients with inhalation burn, severe facial injury, and penetrating neck trauma who come to the ED during off hours and require advanced airway management.

This study presents objective evidence for an association between presentation time and ETI-related adverse events in injured patients with a predicted difficult airway. Off-hour presentation was significantly associated with an increased risk of ETI-related adverse events, independent of anesthesiology specialty, ISS, and use of paralytic agents. There are three plausible explanations for this finding. First, given that DAM in severely injured patients requires the simultaneous assistance of several appropriately trained caregivers, the reduced staffing level during off hours may negatively affect airway management performance. In the DAM algorithms advocated by several professional anesthesiology societies [1–3], the common first step is the “call for help.” Limited help during nights and weekdays is one of the greatest disadvantages in DAM and the resulting scenario is very unlike that during business hours. Second, there is a general trend that medical providers who work in hospitals during off hours have less seniority [40, 41] and experience than those who work during business hours. Supervision by experienced clinicians also tends to be less available [6]. Schmidt et al [33] recently reported that supervision by expert providers was associated with a decreased incidence of complications during emergent ETIs. A national survey performed in the UK [16] found that at least some of the fatal airway management complications in EDs or ICUs occurred during off hours and were associated with a lack of adequate manpower and supervision. Third, the mood, cognitive performance, and psychomotor function of medical staff can be impaired during off hours by fatigue and disrupted circadian rhythms [7, 42]. We and other researchers [5, 43] previously found that severe surgical complications, including missed major trauma and postoperative hemorrhage, increased during off hours. Taken together, these data suggest the need for extreme care when the management of a difficult airway during off hours is unavoidable.

In the present study, 35.0 % of the injured patients with a predicted DAM scenario experienced severe ETI-related adverse events. This high rate is comparable to the rates determined in previous studies conducted in a different setting or patient population [15, 32]. To reduce life-threatening complications, a standard operating procedure for ETI [19] should be implemented. Jaber et al. [4] recently reported that after the introduction of an “intubation bundle” including the routine use of capnometry, ETI-related complications in critically ill patients were significantly reduced. According to Berkow et al. [44], after implementation of a comprehensive DAM program including standardized equipment preparations, the need for emergency surgical airway decreased. In many Japanese EDs, including our own, procedural preferences for ETI varies greatly [17], and such a standardized procedure is lacking.

Consistent with previous reports [45, 46], severe trauma presentations were common in our ED during off hours. As a result, the ED staff were frequently confronted with difficult airways when human resources were suboptimal. To address this issue, the system for hospital-wide DAM coverage during nights and weekends must be improved, including changing physician call schedules to ensure consistent care [46]. The UK national survey also proposed [16] that every hospital should have in-house DAM backup coverage during off hours.

To date, few studies have examined the effects of off-hour presentation on ETI-related complications, and the results have been conflicting [11–13]. In their retrospective observational study of a single pediatric ICU, Carroll et al. [11] found that ETI-associated adverse events were significantly increased during off hours. In a study of nine ICUs in France, Jaber et al. [13] found that the occurrence of life-threatening ETI complications did not differ between off hours and business hours. Martin et al [12] analyzed their university-hospital registry of 3423 emergency airway management situations occurring outside the operating room and found no evidence of an association between off hours and airway-related complications. Our study focused on DAM in trauma patients, including those with inhalational burn, comminuted facial trauma, and penetrating neck injuries. Because airway management of these high-risk population requires the simultaneous involvement of several medical caregivers, the consequences of reduced staffing during off hours may be more apparent in these situations. A specialized trauma care system, including the concentration of patients, medical staff, and other human resources into level I trauma centers [47], has yet to be implemented throughout Japan. For example, most Japanese community hospitals, including our own, do not comply with the American College of Surgeons standards for a level I [47], or even a level II, trauma center [47]. The discrepancies between our findings and those of previous studies may reflect differences in the studied patient population, the medical setting, standard operating procedure for ETI, the healthcare systems, or interactions between these factors.

Based on the findings of this study, we speculate that the risk of severe ETI complications during off hours can be increased in other high-risk patients, such as those with epiglottitis. The outcome of trauma patients with a difficult airway who undergo ETI at a level I trauma centers within a more fully developed trauma care system equipped with a high concentration of medical resources may differ. Our observations should encourage further studies of other high-risk populations or other settings.

### Limitations and strengths

This study had four major limitations. First, its retrospective nature may have increased the risk of bias, including self-reporting, recall, and diagnostic biases. Despite the use of structured ED record and quality assurance databases that captured all severe adverse events occurring in the ED, ETI complications may have been missed, underestimated, or misclassified. To mitigate this limitation, in the analysis of ETI-related adverse events information on the presentation time was masked. Because alternative techniques such as video laryngoscopy and fiber-optic intubation differ substantially from direct laryngoscopy, this population was excluded from the analysis. Although the excluded population was relatively small (about 15 %, Fig. 1) and the backgrounds similar, there is a possibility of selection bias. Second, as with any observational study, an association between presentation time and ETI complications may be confounded by other factors. Although adjustments were made for previously known confounding factors using a logistic regression model, there may have been other, unmeasured confounders. For example, whether the ETI was truly emergent or preventive may have affected both ETI success rate and ETI-related adverse events [11]. However, our database did not record this variable. That the adjustment for ETI complications was incomplete was also possible, because our sample size was small and prevented more rigorous adjustment. Third, while our ED is typical of a Japanese community ED, as with any single-center study it may not be possible to extrapolate our findings to other medical institutions, especially those abroad or that are well-resourced. Fourth, off-duty ED physicians are not required to remain in house, but they often stay until late at night. This fact was not considered in our study, as our database did not include information regarding the true number of participating ED physicians, but it may have affected care and outcomes. However, this study also had several strengths. First, it provided objective information on an association between off–hour presentation and ETI complications in trauma patients with inhalation burns, comminuted facial traumas, and penetrating neck injuries. To the best of our knowledge, this study is the first to analyze this relationship in these important subsets and thus to provide relevant information regarding DAM in the trauma population. Second, because we used pre-specified ED records and our department has a rigorous peer review process supervised by its director, there were no missing data. We therefore believe that our study provides an accurate depiction of advanced airway management in trauma patients with a predicted difficult airway. The information presented herein should be taken into account by medical providers who manage trauma patients or participate in DAM, as well as by hospital administrators and policy-makers.

## Conclusion

In the preset study, conducted in a Japanese community ED, off-hour presentation was independently associated with ETI-related adverse events in trauma patients with a predicted difficult airway, including those with inhalation burns, comminuted facial traumas, and penetrating neck injuries. Our data highlight the need for care providers who participate in emergency airway management to be aware of this disadvantage and to therefore be more vigilant regarding the potential for respiratory failure in ED patients treated during nights and weekends. They also imply the need to improve hospital-wide DAM coverage systems to provide consistent care during nights and weekends.

## Additional file

Additional file 1: Table S1. The ED census from 2007 to 2016. (DOCX 14 kb)

## Abbreviations

AIS: Abbreviated Injury Scale Score
CI: Confidence interval
DAM: Difficult airway management
ED: Emergency department
ETI: Endotracheal intubation
ICU: Intensive care unit
ISS: Injury Severity Score
OR: Odds ratio
Ps: Probability of survival
RTS: Revised Trauma Score

## References

[1] Japanese Society of Anesthesiologists (2014). JSA airway management guideline 2014: to improve the safety of induction of anesthesia. J Anesth.

[2] Apfelbaum JL, Hagberg CA, Caplan RA, Blitt CD, Connis RT, Nickinovich DG, Hagberg CA, Caplan RA, Benumof JL, Berry FA, Blitt CD, Bode RH, Cheney FW, Connis RT, Guidry OF, Nickinovich DG, Ovassapian A, American Society of Anesthesiologists Task Force on Management of the Difficult Airway (2013). Practice guidelines for management of the difficult airway: an updated report by the American Society of Anesthesiologists Task Force on Management of the Difficult Airway. Anesthesiology.

[3] Henderson JJ, Popat MT, Latto IP, Pearce AC, Difficult Airway Society (2004). Difficult Airway Society guidelines for management of the unanticipated difficult intubation. Anaesthesia.

[4] Jaber S, Jung B, Corne P, Sebbane M, Muller L, Chanques G, Verzilli D, Jonquet O, Eledjam JJ, Lefrant JY (2010). An intervention to decrease complications related to endotracheal intubation in the intensive care unit: a prospective, multiple-center study. Intensive Care Med.

[5] Ono Y, Ishida T, Iwasaki Y, Kawakami Y, Inokuchi R, Tase C, Shinohara K (2015). The off-hour effect on trauma patients requiring subspecialty intervention at a community hospital in Japan: a retrospective cohort study. Scand J Trauma Resusc Emerg Med.

[6] Saposnik G, Baibergenova A, Bayer N, Hachinski V (2007). Weekends: a dangerous time for having a stroke?. Stroke.

[7] Johnson J (2008). The increased incidence of anesthetic adverse events in late afternoon surgeries. AORN J.

[8] Peberdy MA, Ornato JP, Larkin GL, Braithwaite RS, Kashner TM, Carey SM, Meaney PA, Cen L, Nadkarni VM, Praestgaard AH, Berg RA, National Registry of Cardiopulmonary Resuscitation Investigators (2008). Survival from in-hospital cardiac arrest during nights and weekends. JAMA.

[9] Kostis WJ, Demissie K, Marcella SW, Shao YH, Wilson AC, Moreyra AE, Myocardial Infarction Data Acquisition System (MIDAS 10) Study Group (2007). Weekend versus weekday admission and mortality from myocardial infarction. N Engl J Med.

[10] Bell CM, Redelmeier DA (2001). Mortality among patients admitted to hospitals on weekends as compared with weekdays. N Engl J Med.

[11] Carroll CL, Spinella PC, Corsi JM, Stoltz P, Zucker AR (2010). Emergent endotracheal intubations in children: be careful if it’s late when you intubate. Pediatr Crit Care Med.

[12] Martin LD, Mhyre JM, Shanks AM, Tremper KK, Kheterpal S (2011). 3,423 emergency tracheal intubations at a university hospital: airway outcomes and complications. Anesthesiology.

[13] Jaber S, Amraoui J, Lefrant JY, Arich C, Cohendy R, Landreau L, Calvet Y, Capdevilla X, Mahamat A, Eledjam JJ (2006). Clinical practice and risk factors for immediate complications of endotracheal intubation in the intensive care unit: a prospective, multiple-center study. Crit Care Med.

[14] Mort TC (2004). Emergency tracheal intubation: complications associated with repeated laryngoscopic attempts. Anesth Analg.

[15] De Jong A, Molinari N, Terzi N, Mongardon N, Arnal JM, Guitton C, Allaouchiche B, Paugam-Burtz C, Constantin JM, Lefrant JY, Leone M, Papazian L, Asehnoune K, Maziers N, Azoulay E, Pradel G, Jung B, Jaber S, AzuRéa Network for the Frida-Réa Study Group (2013). Early identification of patients at risk for difficult intubation in the intensive care unit: development and validation of the MACOCHA score in a multicenter cohort study. Am J Respir Crit Care Med.

[16] Cook TM, Woodall N, Harper J, Benger J, Fourth National Audit Project (2011). Major complications of airway management in the UK: results of the Fourth National Audit Project of the Royal College of Anaesthetists and the Difficult Airway Society. Part 2: intensive care and emergency departments. Br J Anaesth.

[17] Hasegawa K, Hagiwara Y, Chiba T, Watase H, Walls RM, Brown DF, Brown CA, Japanese Emergency Medicine Research Alliance (2012). Emergency airway management in Japan: interim analysis of a multi-center prospective observational study. Resuscitation.

[18] O’Malley RN, O’Malley GF, Ochi G (2001). Emergency medicine in Japan. Ann Emerg Med.

[19] Sherren PB, Tricklebank S, Glover G (2014). Development of a standard operating procedure and checklist for rapid sequence induction in the critically ill. Scand J Trauma Resusc Emerg Med.

[20] Baker SP, O’Neill B, Haddon W, Long WB (1974). The injury severity score: a method for describing patients with multiple injuries and evaluating emergency care. J Trauma.

[21] Baker SP, O’Neill B (1976). The injury severity score: an update. J Trauma.

[22] Champion HR, Sacco WJ, Carnazzo AJ, Copes W, Fouty WJ (1981). Trauma score. Crit Care Med.

[23] Champion HR, Sacco WJ, Copes WS, Gann DS, Gennarelli TA, Flanagan ME (1989). A revision of the Trauma Score. J Trauma.

[24] Champion HR, Sacco WJ, Hunt TK (1983). Trauma severity scoring to predict mortality. World J Surg.

[25] Boyd CR, Tolson MA, Copes WS (1987). Evaluating trauma care: the TRISS method. Trauma Score and the Injury Severity Score. J Trauma.

[26] Champion HR, Copes WS, Sacco WJ, Lawnick MM, Keast SL, Bain LW, Flanagan ME, Frey CF (1990). The Major Trauma Outcome Study: establishing national norms for trauma care. J Trauma.

[27] Deyo RA, Cherkin DC, Ciol MA (1992). Adapting a clinical comorbidity index for use with ICD-9-CM administrative databases. J Clin Epidemiol.

[28] Charlson ME, Pompei P, Ales KL, MacKenzie CR (1987). A new method of classifying prognostic comorbidity in longitudinal studies: development and validation. J Chronic Dis.

[29] Helling TS, Nelson PW, Shook JW, Lainhart K, Kintigh D (2003). The presence of in-house attending trauma surgeons does not improve management or outcome of critically injured patients. J Trauma.

[30] Ono Y, Kikuchi H, Hashimoto K, Sasaki T, Ishii J, Tase C, Shinohara K (2015). Emergency endotracheal intubation-related adverse events in bronchial asthma exacerbation: can anesthesiologists attenuate the risk?. J Anesth.

[31] Walls RM, Brown CA, Bair AE, Pallin DJ, NEAR II Investigators (2011). Emergency airway management: a multi-center report of 8937 emergency department intubations. J Emerg Med.

[32] Hasegawa K, Shigemitsu K, Hagiwara Y, Chiba T, Watase H, Brown CA, Brown DF, Japanese Emergency Medicine Research Alliance Investigators (2012). Association between repeated intubation attempts and adverse events in emergency departments: an analysis of a multicenter prospective observational study. Ann Emerg Med.

[33] Schmidt UH, Kumwilaisak K, Bittner E, George E, Hess D (2008). Effects of supervision by attending anesthesiologists on complications of emergency tracheal intubation. Anesthesiology.

[34] Lockey D, Crewdson K, Weaver A, Davies G (2014). Observational study of the success rates of intubation and failed intubation airway rescue techniques in 7256 attempted intubations of trauma patients by pre-hospital physicians. Br J Anaesth.

[35] Breckwoldt J, Klemstein S, Brunne B, Schnitzer L, Arntz HR, Mochmann HC (2012). Expertise in prehospital endotracheal intubation by emergency medicine physicians—comparing ‘proficient performers’ and ‘experts’. Resuscitation.

[36] Tayal VS, Riggs RW, Marx JA, Tomaszewski CA, Schneider RE (1999). Rapid-sequence intubation at an emergency medicine residency: success rate and adverse events during a two-year period. Acad Emerg Med.

[37] Sagarin MJ, Barton ED, Chng YM, Walls RM, National Emergency Airway Registry Investigators (2005). Airway management by US and Canadian emergency medicine residents: a multicenter analysis of more than 6,000 endotracheal intubation attempts. Ann Emerg Med.

[38] Li J, Murphy-Lavoie H, Bugas C, Martinez J, Preston C (1999). Complications of emergency intubation with and without paralysis. Am J Emerg Med.

[39] Combes X, Andriamifidy L, Dufresne E, Suen P, Sauvat S, Scherrer E, Feiss P, Marty J, Duvaldestin P (2007). Comparison of two induction regimens using or not using muscle relaxant: impact on postoperative upper airway discomfort. Br J Anaesth.

[40] Thorpe KE (1990). House staff supervision and working hours. Implications of regulatory change in New York State. JAMA.

[41] McKee M, Black N (1992). Does the current use of junior doctors in the United Kingdom affect the quality of medical care?. Soc Sci Med.

[42] Olson EJ, Drage LA, Auger RR (2009). Sleep deprivation, physician performance, and patient safety. Chest.

[43] Bendavid E, Kaganova Y, Needleman J, Gruenberg L, Weissman JS (2007). Complication rates on weekends and weekdays in US hospitals. Am J Med.

[44] Berkow LC, Greenberg RS, Kan KH, Colantuoni E, Mark LJ, Flint PW, Corridore M, Bhatti N, Heitmiller ES (2009). Need for emergency surgical airway reduced by a comprehensive difficult airway program. Anesth Analg.

[45] Carr BG, Reilly PM, Schwab CW, Branas CC, Geiger J, Wiebe DJ (2011). Weekend and night outcomes in a statewide trauma system. Arch Surg.

[46] Carmody IC, Romero J, Velmahos GC (2002). Day for night: should we staff a trauma center like a nightclub?. Am Surg.

[47] American College of Surgeons Committee on Trauma. Resources for optimal care of the injured patient 2014 https://www.facs.org/~/media/files/quality%20programs/trauma/vrc%20resources/resources%20for%20optimal%20care.ashx. Accessed 25 Dec 2014.

