# Supplementary material for: Association between off-hour presentation and endotracheal-intubation-related adverse events in trauma patients with a predicted difficult airway: A historical cohort study at a community emergency department in Japan
Source: Scand J Trauma Resusc Emerg Med. 2016 Aug 30;24(1):106. doi: 10.1186/s13049-016-0296-2 (PMC5006537; doi:10.1186/s13049-016-0296-2)
Supplement: Additional file 1: Table S1. — The ED census from 2007 to 2016. (DOCX 14 kb) [file 13049_2016_296_MOESM1_ESM.docx]

| **Number of patients brought to the ED** |  |
| --- | --- |
| All | 50,764 |
| Trauma etiology | 11,458 |
| Non-trauma etiology | 39,306 |
| **Occurrence of ETI** | **% (n)** |
| All | 6.7 (3,407/50,764) |
| Trauma etiology | 6.1 (702/11,458) |
| Non-trauma etiology | 6.9 (2,705/39,306) |
| **Overall ETI success rate** | **% (n)** |
| All | 99.6 (3,394/3,407) |
| Trauma etiology^a^ | 98.9 (694/702) |
| Non-trauma etiology^b^ | 99.8 (2,700/2,705) |

**Table S1** The ED census from 2007 to 2016

^a^Four patients with inhalation burn, two with penetrating neck injury, and two with comminuted facial trauma (AIS Face ≥3) received emergency surgical airway after failed ETI attempts.

^b^Four patients with advanced laryngeal cancer and one with epiglottitis received emergency surgical airway after failed ETI attempts.

AIS, Abbreviated Injury Scale Score; ED, emergency department; ETI, endotracheal intubation.
